# Simultaneous bilateral total knee arthroplasty with and without patellar resurfacing using a zirconium ceramic femoral component demonstrated equivalent short‐term outcomes: A prospective randomised clinical trial

**DOI:** 10.1002/jeo2.70436

**Published:** 2025-10-06

**Authors:** Hiroki Katagiri, Hideyuki Koga, Ryusuke Saito, Yusuke Nakagawa, Tomomasa Nakamura, Ichiro Sekiya, Toshifumi Watanabe

**Affiliations:** ^1^ Department of Joint Surgery and Sports Medicine, Graduate School of Medical and Dental Sciences Institute of Science Tokyo Tokyo Japan; ^2^ Department of Orthopaedic Surgery Dokkyo Medical University Saitama Medical Center Koshigaya Japan; ^3^ Department of Orthopaedic Surgery Institute of Science Tokyo Hospital Tokyo Japan; ^4^ Center for Stem Cell and Regenerative Medicine Institute of Science Tokyo Tokyo Japan

**Keywords:** patellar non‐resurfacing, patellar resurfacing, simultaneous bilateral total knee arthroplasty, zirconium ceramic femoral component

## Abstract

**Purpose:**

The optimal management of the patella during total knee arthroplasty (TKA) has not been updated with the development of prosthesis. The purpose of this prospective randomised clinical trial was to compare the physical findings and patient‐reported outcome measures between knees with patellar resurfacing and those without patellar resurfacing in the same patients undergoing simultaneous bilateral TKA using a contemporary zirconium ceramic femoral component.

**Methods:**

Forty patients (80 knees) scheduled for simultaneous bilateral primary TKA due to osteoarthritis received patellar resurfacing on one side knee indicated by randomisation (either left or right) and patellar non‐resurfacing on the opposite side. A posterior‐stabilised prosthesis with the contemporary zirconium ceramic patella‐friendly femoral component (ACTIYAS Kyocera, Kyoto, Japan) was used with the measured resection technique with mechanical alignment in all cases. All patients were followed for a minimum of 2 years. Knee Society knee score and function score, the 2011 Knee Society score, numerical rating scale for pain, patient overall assessment of the knee (0 being “worst”−100 being “normal”), and symptom around the patella were compared between the two groups.

**Results:**

There were no significant differences in postoperative objective knee indicators, postoperative patient‐reported outcome measures, and postoperative examination of the patellofemoral joint between the knees that received patellar resurfacing and patellar non‐resurfacing, without baseline differences. Patient overall assessment of the knee averaged 86.3 in the patella resurfacing group while 84.2 in the non‐resurfacing group (*p* = 0.142). Objective knee indicators and the patient‐reported outcome measures increased significantly after surgery in both groups. Secondary resurfacing and reoperation were not required for any patellar resurfacing or non‐resurfacing knees.

**Conclusion:**

Excellent physical findings and patient‐reported outcome measures were observed following TKA with the contemporary zirconium ceramic femoral component, regardless of whether patellar resurfacing was performed, in the short term.

**Level of Evidence:**

Level II, lower quality randomised trials.

**Abbreviations**
2011 KSS: the 2011 Knee Society score
FTA: femorotibial angles
KS: knee society knee score
PS: posterior‐stabilised
TKA: total knee arthroplasty

## INTRODUCTION

The optimal management of the patella during total knee arthroplasty (TKA) has not been updated [17, 37], despite continuous technological and procedural advances in the field [5, 11, 20, 36]. The disease burden of differing complication profiles associated with patellar resurfacing and non‐resurfacing remains unclear [31]. Early‐generation patellar prosthesis have been associated with various complications, such as patella fracture (reported incidence rate: 0.5%–5.2%), polyethylene wear (1.6–5.0%), polyethylene fracture (0.5%–3.8%) and component loosening (0.4%–4.0%) [4, 8, 22, 26]. In response to these complications, femoral prostheses compatible with patellar resurfacing have been designed. These designs feature more accommodating trochlear characteristics, such as an asymmetrically shaped, anatomically designed, non‐dysplastic trochlear groove with a suitably sized lateral facet that is not excessively high [16, 23].

On the other hand, one of the predominant issues with patellar non‐resurfacing is anterior knee pain [24]. To address this problem, the development of materials with high biocompatibility has been pursued. A densitometry study indicated that postoperative correct patella loading and kinematics lead to a recovery in bone density of the patella following patellar non‐resurfacing TKA, with a significant improvement in knee functional scores [6]. Among these, zirconia ceramic stands out due to its superior wettability, greater surface smoothness, and minimal negative effects on cell viability [29, 35]. A study on the biocompatibility of zirconia ceramic bearings against articular cartilage using a tribological bioreactor demonstrated that cartilage in contact with zirconia ceramic had 17% fewer dead cells and 30% less collagen debris release, specifically hydroxyproline, compared to cartilage in contact with cobalt‐chromium alloy [35]. A prosthesis compatible with both patellar resurfacing and non‐resurfacing (ACTIYAS Kyocera, Kyoto, Japan) was developed in 2010, featuring a zirconium ceramic femoral component with contemporary accommodating trochlear characteristics.

The purpose of this study was to compare the physical findings and patient‐reported outcome measures between knees with patellar resurfacing and those without patellar resurfacing in the same patients undergoing simultaneous bilateral TKA with a zirconium ceramic contemporary femoral component. A side‐by‐side comparison of knees with patellar resurfacing and non‐resurfacing in the same patients during simultaneous bilateral TKA can assess patient‐reported outcome measures without affecting patient‐response styles, behaviours, and temperament [2]. The underlying hypothesis for this study was that there were no significant differences in physical findings and patient‐reported outcome measures between knees undergoing patellar resurfacing and those without resurfacing.

## METHODS

### Enrolment

**Figure 1:**
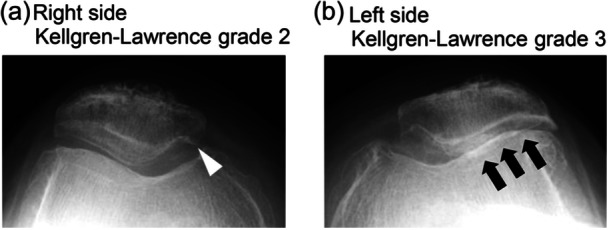
Representative X‐ray images showing asymmetry in patellofemoral joint degeneration on skyline view. (a) Right knee demonstrating Kellgren–Lawrence grade 2 changes, characterised by definite osteophyte formation (white arrow head). (b) Left knee demonstrating Kellgren–Lawrence grade 3 changes, with definite joint space narrowing (black arrows).

**Figure 2:**
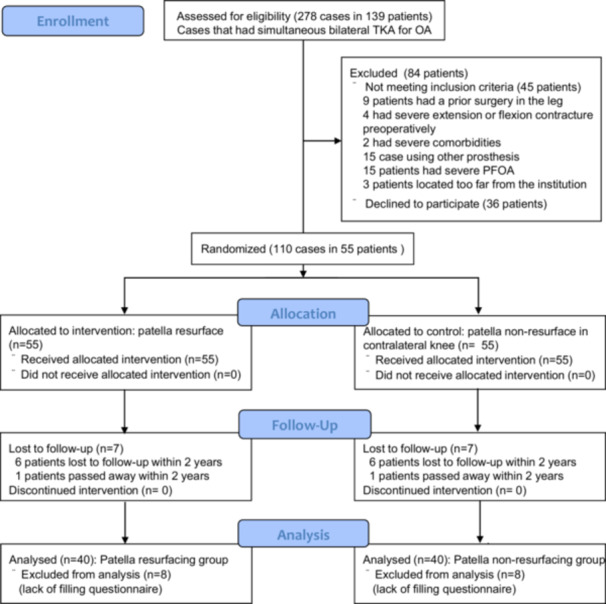
Study flow chart as per the CONSORT 2010 guidelines. OA, osteoarthritis; PFOA, patellofemoral osteoarthritis; TKA, total knee arthroplasty.

This study was designed as per the CONSORT 2010 guidelines [27]. The minimum sample size required to detect a significant difference exceeding the minimal clinically important difference of 2.0 [25] for the numerical pain rating scale was calculated with an *α* of 0.05 and *β* of 0.8. Using a previously reported standard deviation of 2.5 [21], the required sample size was determined to be 37 participants per group. A total of 55 patients were enroled to allow for loss of follow‐up. From September 2013 to July 2016, patients who received single‐staged bilateral TKA for osteoarthritis were assessed for eligibility. The exclusion criteria were as follows: (1) patients who had undergone prior leg surgery; (2) patients who had severe extension (>30°) or flexion (<90°) contracture preoperatively; (3) patients who had severe comorbidities; (4) patients with other prosthesis, large bony defects, knee joint instability, additional metal augment or restrictive prosthesis; (5) patients with severe patellofemoral osteoarthritis (marked narrowing of the joint space: Kellgren–Lawrence Grade 4) or those with asymmetry in patellofemoral joint degeneration, defined as a difference of more than one grade on the Kellgren–Lawrence grading scale in the skyline view (Figure 1); [10] and (6) patients located too far from the institution. Eligible participants were recruited for the randomised control study, the process was explained, and the participants signed an informed consent form. Participants were free to withdraw at any time. After excluding 139 patients based on the exclusion criteria, 55 patients were enroled in the study (Figure 2).

**Figure 3:**
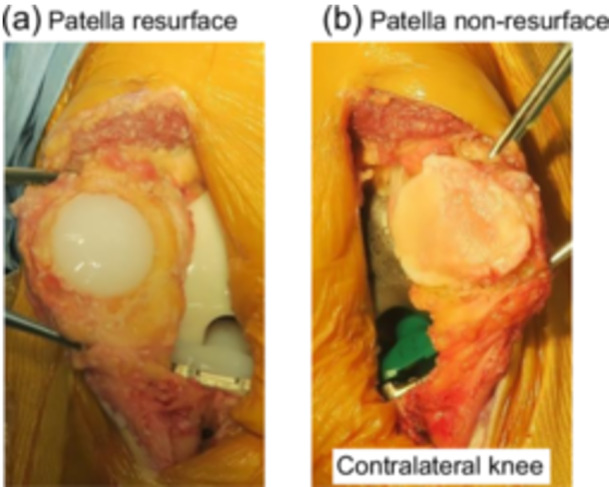
(a) The right knee underwent implant insertion with patellar resurfacing, as indicated by randomisation (resurfacing group). (b) The left knee underwent implant insertion without patellar resurfacing (non‐resurfacing group).

A random number generator computer function was used, and the generated numbers were placed into envelopes. Randomisation was accomplished by opening a randomly selected envelope in the operation room. One knee indicated by randomisation, either left or right, received treatment with the patellar resurfacing (resurfacing group), while the contralateral knee received treatment without patellar resurfacing (non‐resurfacing group) (Figure 3). All patients had one patellar resurfacing and the contralateral patellar non‐resurfacing. This study was approved by the institutional review board of Institute of Science Tokyo Hospital (research protocol identification number: 1547).

### Surgery

All procedures were performed using the measured resection technique with mechanical alignment and tourniquet control under general anaesthesia by seven board‐certified orthopaedic surgeons according to the manufacturer's instructions [12, 13]. A posterior‐stabilised (PS) prosthesis (ACTIYAS Kyocera, Kyoto, Japan), the zirconium ceramic contemporary patella‐friendly femoral component, was used in all cases [32]. In brief, a standard midvastus approach was used. Half the infrapatellar fat pad was resected to expose the lateral plateau of tibia, while preserving the other half. Measured resection was performed based on the anatomical landmarks. The distal femurs were resected to an implant thickness of 9 mm from the less worn area using an intramedullary rod, and then trimmed perpendicularly to the mechanical axis of the femur. Femoral rotation was aligned parallel to the femoral epicondylar axis, determined by the rotation angle between the posterior condylar axis and the femoral epicondylar axis based on measurements taken from preoperative CT scans. The proximal tibia was resected to a thickness of 10 mm from the less worn area of the lateral tibial plateau and trimmed perpendicularly to the mechanical axis of the tibia using an extramedullary alignment rod. Patellar osteophyte removal was completed in both the patellar resurfacing and the contralateral non‐resurfacing knees. Patellar denervation were not performed in both groups. In the patellar resurfacing knee, the patellar thickness was measured by calipers, and the patellar was resected to the same thickness as the patellar prosthesis height. Patellar tracking was assessed by the no thumb test. All prostheses were fixed with cement. All patients were managed with the same postoperative rehabilitation protocol, which did not vary by sides. Patients were instructed on a range of motion exercises, and full weight bearing and walking exercises with a walker were started on the day after surgery.

### Clinical evaluations

Age at surgery, sex, weight, height, and body mass index were recorded preoperatively. Preoperative and postoperative femorotibial angles (FTA) were measured on long‐leg weight‐bearing anteroposterior radiographs by a single orthopaedic surgeon (R.S.) with more than 15 years of experience in orthopaedic surgery. Extension and flexion range of the knee joint were measured using a goniometer before surgery and at final follow‐up (at least 2 years after surgery). Knee Society knee score (KS) and functional score [28] were documented by the attending surgeon before surgery and 2 years after surgery. The substantial clinical benefit score for KS was used, where 40 points correlated to substantial clinical benefit [15]. Before surgery and at the final follow‐up appointment, the following scores were completed on each side of legs by each patient: subjective section of the 2011 Knee Society score (2011 KSS); numerical pain rating scale for walking, rest, first movement, and stair gait; patient overall assessment of the knee ('0' 'worst'−'100' 'being normal'); [19] and instability during stair gait ('0' 'no instability'−'10' 'worst instability'). Postoperative patellar grind test was carried out for crepitus and pain assessment, and sound around the patella was documented by the attending surgeon at final follow‐up.

### Statistical analysis

Statistical analyses were performed using IBM SPSS Statistics for Windows, version 25.0 (IBM Corp., Armonk, New York). Positive rate of postoperative patellar grind test crepitus, postoperative patellar grind test pain, postoperative sound around the patella were compared between the two groups by Fisher's exact test. The other items were compared between two groups by paired *t*‐tests, after confirming normality using a histogram and equal variance by *F*‐tests. *p* < 0.05 was considered statistically significant. Data were expressed as mean and standard deviation values or as mean values with minimum and maximum values.

### Results

The mean patient follow‐up period was 47 months (24–72 months), with no re‐operation required in this period. Objective knee indicators, extension range of the knee joint, and FTA significantly improved post surgically, while flexion range of the knee joint did not change in either group. ks increased significantly after surgery, with values exceeding the substantial clinical benefit score of 40 points in both groups (Table 1). Improvement of KS, in four patients in the patella resurfacing group and five patients in the non‐resurfacing group, was less than the substantial clinical benefit score. There were no significant differences in the number of patients who did not reach the substantial clinical benefit score between the two groups. There were no significant differences in any indicators before surgery or at the final follow‐up between the patellar resurfacing and non‐resurfacing groups.

**Table 1:** Preoperative and postoperative objective knee indicators.

		Patella resurfacing group				Patella non‐resurfacing group			Between groups	
	Pre operation	Post operation	Post minus pre	Pre‐Post *p*‐value	Pre operation	Post operation	Post minus pre	*p*‐value	Pre‐op *p*‐value	Post‐op *p*‐value
Number	40	40	40		40	40	40			
Extension range of the knee (°)	−6.0 ± 5.9 [0 to −20]	−1.8 ± 4.0 [3 to −15]	4.3 ± 5.1 [−3 to 20]	**<0.001**	−6.7 ± 6.3 [0 to −20]	−1.8 ± 3.6 [2 to −15]	5.0 ± 6.0 [−5 to 20]	**<0.001**	0.770	0.915
Flexion range of the knee(°)	125.5 ± 13.3 [95–145]	128.6 ± 8.8 [110–145]	3.1 ± 11.0 [−15 to 35]	0.079	125.0 ± 14.8 [80–145]	128.1 ± 8.5 [110–145]	3.1 ± 11.4 [−25 to 35]	0.090	0.198	0.323
Femorotibial angle (°)	185.0 ± 5.1 [170–195]	173.8 ± 2.6 [169–179]	−11.2 ± 5.2 [−20 to 5]	**<0.001**	185.4 ± 4.4 [176–196]	174.4 ± 2.1 [170–178]	−11.0 ± 4.5 [−23 to 0]	**<0.001**	0.561	0.200
Knee Society knee score	42.7 ± 11.6 [10–65]	96.5 ± 6.9 [65–100]	52.8 ± 13.4 [24–87]	<0.001	42.9 ± 10.0 [17–60]	95.3 ± 6.2 [68–100]	52.4 ± 12.0 [21–80]	<0.001	0.925	0.693

*Note*: Values with brackets are mean with standard deviation and range.

Of the patient‐reported outcome measures, patient overall assessment of the knee, numerical pain rating scale scores, and subjective parts of the 2011 KSS (symptoms, patient satisfaction, and patient expectation) improved post surgically in both groups (Table 2). There were no significant differences in any patient‐reported outcome measures before surgery and at the final follow‐up between the patellar resurfacing and non‐resurfacing groups. Knee Society functional score and all functional activity scores in the 2011 KSS significantly increased after surgery (Table 3). There were no significant differences between the patellar resurfacing and non‐resurfacing groups in the positive rate of crepitus, pain during the patellar grind test, or sounds around the patella (Table 4).

**Table 2:** Preoperative and postoperative patient‐reported outcome measures.

		Patella resurfacing group				Patella non‐resurfacing group			Between groups	
	Preoperation	Post operation	Post minus pre	Pre Post *p*‐value	Pre operation	Post operation	Post minus pre	*p*‐value	Pre‐op *p*‐value	Post‐op *p*‐value
Number	40	40	40		40	40	40			
Patient overall assessment of the knee	28.8 ± 21.8 [0–70]	86.3 ± 16.0 [30–100]	57.6 ± 25.7 [10–100]	**<0.001**	27.4 ± 21.2 [0–80]	84.2 ± 16.5 [40–100]	56.9 ± 26.2 [10–100]	**<0.001**	0.628	0.142
Numerical pain rating scale										
Walking	6.2 ± 2.3 [1–10]	1.2 ± 1.8 [0–8]	‐5.0 ± 2.5 [−10 to −1]	**<0.001**	6.1 ± 2.3 [1–10]	1.3 ± 1.8 [0–7]	−4.8 ± 2.8 [−10 to 2]	**<0.001**	0.650	0.559
Rest	3.8 ± 2.6 [0–10]	0.9 ± 1.6 [0–8]	−3.0 ± 2.5 [−10 to 1]	**<0.001**	3.9 ± 2.7 [0–10]	0.8 ± 1.4 [0–5]	−3.2 ± 2.4 [−10 to 0]	**<0.001**	0.686	0.917
First movement	6.0 ± 2.6 [1–10]	1.3 ± 1.6 [0–8]	−4.8 ± 2.7 [−10 to 0]	**<0.001**	6.0 ± 2.7 [1–10]	1.4 ± 1.5 [0–6]	−4.7 ± 3.0 [−10 to 1]	**<0.001**	1.000	0.586
Stair gait	6.6 ± 2.9 [0–10]	2.0 ± 2.1 [0–8]	−4.5 ± 2.9 [−10 to 2]	**<0.001**	6.3 ± 2.6 [0–10]	2.1 ± 2.1 [0–8]	−4.2 ± 3.0 [−10 to 5]	**<0.001**	0.357	0.635
2011 Knee Society score										
Symptoms (0–25)	6.0 ± 5.3 [0– 18]	20.4 ± 5.3 [5–25]	14.5 ± 7.5 [−2 to 25]	**<0.001**	5.7 ± 5.0 [0–17]	20.3 ± 5.0 [7–25]	14.6 ± 6.8 [3–25]	**<0.001**	0.657	0.486
Patient satisfaction (40)	10.7 ± 5.0 [0–20]	26.5 ± 7.7 [12–40]	15.9 ± 8.7 [0–34]	**<0.001**	11.0 ± 4.4 [0–20]	26.6 ± 8.2 [12–40]	15.6 ± 9.0 [2–36]	**<0.001**	0.596	0.928
Patient expectation (15)	13.8 ± 4.0 [0–30]	10.1 ± 2.5 [6–15]	−3.7 ± 4.3 [−21 to 7]	**<0.001**	13.7 ± 3.7 [0–30]	10.0 ± 2.7 [5–15]	−3.7 ± 4.1 [−21 to5]	**<0.001**	0.291	0.822

*Note*: Values with brackets are mean with standard deviation and range.

**Table 3:** Preoperative and postoperative functional activity scores.

	Pre operation	Post operation	*p*‐value
Number	40	40	
Knee Society functional score	44.2 ± 17.1 [0–80]	78.0 ± 21.2 [0–100]	**<0.001**
2011 Knee Society score			
Walking and standing (30)	10.7 ± 6.7 [−4 to 24]	21.3 ± 7.5 [1–30]	**<0.001**
Standard activities (30)	11.4 ± 4.8 [0–25]	23.3 ± 5.6 [8–30]	**<0.001**
Advanced activities (25)	4.7 ± 4.2 [0–16]	12.7 ± 6.6 [0–22]	**<0.001**
Discretionary knee activities (15)	4.7 ± 3.7 [0–12]	8.2 ± 6.0 [0–15]	**0.001**
Total functional activities (100)	31.0 ± 15.7 [0–74]	65.4 ± 21.0 [0–54]	**<0.001**

*Note*: Values with brackets are mean with standard deviation and range.

**Table 4:** Postoperative examination of the patellofemoral joint.

	Patella resurface group	Patella non‐resurface group	*p*‐value
Number	40	40	
Positive rate of crepitus with patellar grind test	5% (2/40)	5% (2/40)	1.000
Positive rate of pain with patellar grind test	15% (6/40)	7.5% (3/40)	0.481
Sound around the patella	17.5% (7/40)	17.5% (7/40)	1.000
Instability during stair gait (0−10)	1.1 ± 1.6 [0–7]	1.4 ± 1.8 [0–7]	0.193

*Note*: Values with brackets are mean with standard deviation and range. Values with brackets parentheses are expressed as number with percentage.

## DISCUSSION

We found no significant differences in objective knee indicators, patient‐reported outcome measures, and examination of the patellofemoral joint between the patellar resurfacing and non‐resurfacing groups after simultaneous bilateral TKA using The ACTIYAS with the zirconium ceramic contemporary femoral component. The ACTIYAS prosthesis features a multi‐radius femoral design, which allows for a more natural knee motion throughout the range of flexion. It incorporates a round‐shaped post‐cam mechanism and a slightly concave symmetrical polyethylene insert, designed to minimise extreme constraint and facilitate smooth femoral rotation and translation [34]. Recent studies have demonstrated that a rounded post‐cam design generates less stress concentration during flexion with rotation and at hyperextension compared to a squared design, which may contribute to improved longevity and stability of the prosthesis [32]. Additionally, the ACTIYAS prosthesis utilises a zirconium ceramic femoral component, known for its enhanced wear resistance and biocompatibility. The design also includes a reduced anterior flange volume, which has been reported to provide favourable clinical outcomes, particularly in patients with preoperative varus alignment undergoing TKA [33].

A meta‐analysis of randomised controlled trials demonstrated no significant difference in the risk ratio for anterior knee pain between patellar resurfacing and non‐resurfacing TKA, with patient satisfaction being similar in both groups [31]. A previous clinical trial using the Scorpio NRG knee prosthesis, which performed one‐stage bilateral TKA with and without patellar resurfacing, similar to the current study, reported that the mean KS after patellar resurfacing was 92.07, which was superior to the non‐resurfacing score of 90.98 [9]. Another clinical trial of one‐stage bilateral TKA with and without patellar resurfacing was conducted using the Lospa prostheses (Corentec Co, Ltd., Seoul, Korea) developed in 2010. These prostheses feature a laterally oriented trochlear groove, a high lateral flange, and a deeper and longer intercondylar notch compared to competitors such as Vanguard (Zimmer Biomet) and Triathlon (Stryker Orthopedics) [14]. That clinical trial showed no differences in the patient‐reported outcome measures or side preferences related to patellar resurfacing. In our one‐stage bilateral TKA clinical trial using the ACTIYAS prosthesis, no significant differences were observed in patient‐reported outcome measures between patellar resurfacing and non‐resurfacing TKA. These findings suggest that the design characteristics of both the ACTIYAS and Lospa prostheses may contribute positively to clinical outcomes, regardless of patellar resurfacing status.

After TKA, 15%–25% of patients are dissatisfied with the outcomes [1, 3]. Giesinger et al. statistically determined cutoff values for identifying successful outcomes as 85.5 for the KS [7]. In our study, 90% of the knees in the patellar resurfacing group and 95% in the patellar non‐resurfacing group showed an increase in the KS above those cutoff values, postoperatively. Improvement of KS in 90% of knees in the patella resurfacing group and 87.5% in the non‐resurfacing group was more than the substantial clinical benefit score of 40 points.

Randomised controlled trials showed that the reoperation rate was higher with patellar non‐resurfacing, while patient satisfaction remained similar in both groups [18, 30]. Clinically, it is difficult to determine the cause of postoperative knee pain. However, surgeons are more likely to recommend reoperation for resurfacing a patella if that option is available. In the current study, the positive rate of postoperative patella crepitus or pain with patellar grind test, sound around the patella, and instability during stair gait did not significantly differ between the two groups. The previous clinical trial of one‐stage bilateral TKA with and without patellar resurfacing showed substantial alleviation of anterior knee pain following TKA, regardless of whether resurfacing was performed [14]. Further double‐blind randomised control trial are needed to resolve the discrepancy of the above results.

There were some limitations in this study. First, the study was limited by the relatively small sample size. The number of subjects exceeded the priori sample size required to detect a significant difference exceeding the minimal clinically important difference for the numerical pain rating scale. Despite this, additional studies with larger sample sizes are warranted to enhance the robustness and generalisability of our findings. Second, the PS prosthesis with the zirconium ceramic contemporary patella‐friendly femoral component was used with the measured resection technique. Each surgical technique, concept, and prosthesis can affect patient‐reported outcome measures. Therefore, these findings should be generalised with caution. Third, while there were no differences in the patient‐reported outcome measures between the two groups and no re‐operation cases during follow‐up, the mean follow‐up period was 47 months. Future study with long‐term follow‐up should be performed. Fourth, the clinical outcomes in this study do not include specific patella‐related scores. However, we assessed the patellofemoral joint using measures such as pain during stair climbing, pain at initial movement after rest, crepitus with the patellar grind test, and instability during stair gait.

## CONCLUSION

Excellent physical findings and patient‐reported outcome measures were observed following TKA with the contemporary zirconium ceramic femoral component, regardless of whether patellar resurfacing was performed, in the short term.

## AUTHOR CONTRIBUTIONS

Hiroki Katagiri conducted the study and completed the final manuscript. Ryusuke Saito collected the data and analysed the data and drafted the manuscript. Hiroki Katagiri, Tomomasa Nakamura and Ichiro Sekiya designed the initial plan and collected the data. Toshifumi Watanabe designed the initial plan, conducted the study and completed the final manuscript. All authors read and approved the final manuscript.

## CONFLICT OF INTEREST STATEMENT

The authors declare no conflicts of interest.

## ETHICS STATEMENT

This study was approved by the Institutional Review Board in Institute of Science Tokyo Hospital (research protocol identification number: 1547). Eligible participants were recruited for the randomised control study, the process was explained, and the participants signed an informed consent form. All study participants provided their full written informed consent for participation in this clinical research prior to the operative procedure. Participants were free to withdraw at any time. The authors declare the consent for publication.

## Data Availability

Raw data were generated at the Institute of Science Tokyo. Derived data supporting the findings of this study are available from the corresponding author [T.W.] on request.

## References

[1] Bierke S , Häner M , Karpinski K , Hees T , Petersen W . Midterm effect of mental factors on pain, function, and patient satisfaction 5 years after uncomplicated total knee arthroplasty. J Arthroplasty. 2020;35:105–111. 31477540 10.1016/j.arth.2019.08.008

[2] Bo Z , Liao L , Zhao J , Wei Q , Ding X , Yang B . Mobile bearing or fixed bearing? A meta‐analysis of outcomes comparing mobile bearing and fixed bearing bilateral total knee replacements. Knee. 2014;21:374–381. 24380804 10.1016/j.knee.2013.10.002

[3] Bourne RB , Chesworth BM , Davis AM , Mahomed NN , Charron KDJ . Patient satisfaction after total knee arthroplasty: who is satisfied and who is not? Clin Orthop Relat Res. 2010;468:57–63. 19844772 10.1007/s11999-009-1119-9 PMC2795819

[4] Chun KA , Ohashi K , Bennett DL , El‐Khoury GY . Patellar fractures after total knee replacement. Am J Roentgenol. 2005;185:655–660. 16120913 10.2214/ajr.185.3.01850655

[5] Corti A , Galante S , Rauch R , Chiappetta K , Corino V , Loppini M . Leveraging transfer learning for predicting total knee arthroplasty failure from post‐operative radiographs. J Exp Orthop. 2024;11:e70097. 39664926 10.1002/jeo2.70097 PMC11633713

[6] Di Martino A , Franceschi F , Papalia R , Marini M , Prossomariti G , Maffulli N , et al. Increased bone mineral density in the non‐resurfaced patella after total knee arthroplasty: a clinical and densitometric study. Surgeon. 2012;10:20–24. 22233553 10.1016/j.surge.2011.01.001

[7] Giesinger JM , Hamilton DF , Jost B , Behrend H , Giesinger K . WOMAC, EQ‐5D and Knee Society Score thresholds for treatment success after total knee arthroplasty. J Arthroplasty. 2015;30:2154–2158. 26160647 10.1016/j.arth.2015.06.012

[8] Gustke KA , Simon P , Meheux CJ . Metal‐backed patella implants in knee arthroplasty: can the past predict the future? J Arthroplasty. 2023;38:S131–S136. 10.1016/j.arth.2023.02.013 36791886

[9] Ha C , Wang B , Li W , Sun K , Wang D , Li Q . Resurfacing versus not‐resurfacing the patella in one‐stage bilateral total knee arthroplasty: a prospective randomized clinical trial. Int Orthop. 2019;43:2519–2527. 31227852 10.1007/s00264-019-04361-7 PMC6848038

[10] Heng HY , Bin Abd Razak HR , Mitra AK . Radiographic grading of the patellofemoral joint is more accurate in skyline compared to lateral views. Ann Transl Med. 2015;3:263. 26605309 10.3978/j.issn.2305-5839.2015.10.33 PMC4630554

[11] Jamali AA , Shekhar A , Dungy D , Stewart SL . Kinematic versus mechanical alignment: a systematic review of systematic reviews and meta‐analyses of randomised controlled trials. J Exp Orthop. 2024;11:e70044. 39478687 10.1002/jeo2.70044 PMC11522918

[12] Katagiri H , Saito R , Shioda M , Jinno T , Watanabe T . Effect of posterior capsular release on intraoperative joint gap mismatch in the mid‐flexion range during posterior‐stabilized total knee arthroplasty. J Orthop Sci. 2024;29:200–206. 36522245 10.1016/j.jos.2022.11.019

[13] Katagiri H , Saito R , Shioda M , Jinno T , Watanabe T . Medial osteophyte resection width correlates with correction of the medio‐lateral component gap imbalance during posterior‐stabilized total knee arthroplasty. Clinical Biomechanics. 2022;100:105803. 36309000 10.1016/j.clinbiomech.2022.105803

[14] Koh IJ , Kim MS , Sohn S , Song KY , Choi NY , In Y . Patients undergoing total knee arthroplasty using a contemporary patella‐friendly implant are unaware of any differences due to patellar resurfacing. Knee Surg Sports Traumatol Arthrosc. 2019;27:1156–1164. 30132051 10.1007/s00167-018-5120-2

[15] Lizaur‐Utrilla A , Gonzalez‐Parreño S , Martinez‐Mendez D , Miralles‐Muñoz FA , Lopez‐Prats FA . Minimal clinically important differences and substantial clinical benefits for Knee Society Scores. Knee Surg Sports Traumatol Arthrosc. 2020;28:1473–1478. 31111184 10.1007/s00167-019-05543-x

[16] Lustig S , Servien E , Batailler C . How to optimize patellar tracking in knee arthroplasty? Orthop Traumatol: Surg Res. 2023;109:103458. 36302447 10.1016/j.otsr.2022.103458

[17] McConaghy K , Derr T , Molloy RM , Klika AK , Kurtz S , Piuzzi NS . Patellar management during total knee arthroplasty: a review. EFORT Open Rev. 2021;6:861–871. 34760286 10.1302/2058-5241.6.200156 PMC8559560

[18] Murray DW , MacLennan GS , Breeman S , Dakin HA , Johnston L , Campbell MK , et al. A randomised controlled trial of the clinical effectiveness and cost‐effectiveness of different knee prostheses: the Knee Arthroplasty Trial (KAT). Health Technol Assess (Rockv). 2014;18:1–235. 10.3310/hta18190 PMC4781565 24679222

[19] O'Connor CM , Ring D . Correlation of Single Assessment Numeric Evaluation (SANE) with other Patient Reported Outcome Measures (PROMs). Arch Bone Jt Surg. 2019;7:303–306. 31448305 PMC6686068

[20] Otten TM , Grimm SE , Ramaekers B , Roth A , Emans P , Boymans T , et al. Forecasting the value of innovation in total knee arthroplasty care: a headroom approach. J Exp Orthop. 2024;11:e70096. 39697990 10.1002/jeo2.70096 PMC11653941

[21] Pinto PR , McIntyre T , Ferrero R , Almeida A , Araújo‐Soares V . Risk factors for moderate and severe persistent pain in patients undergoing total knee and hip arthroplasty: a prospective predictive study. PLoS One. 2013;8:e73917. 24058502 10.1371/journal.pone.0073917 PMC3772812

[22] Putman S , Boureau F , Girard J , Migaud H , Pasquier G . Patellar complications after total knee arthroplasty. Orthop Traumatol: Surg Res. 2019;105:S43–S51. 29990602 10.1016/j.otsr.2018.04.028

[23] Roussot MA , Haddad FS . The evolution of patellofemoral prosthetic design in total knee arthroplasty: how far have we come? EFORT Open Rev. 2019;4:503–512. 31538000 10.1302/2058-5241.4.180094 PMC6719608

[24] Russell RD , Huo MH , Jones RE . Avoiding patellar complications in total knee replacement. Bone Jt J. 2014;96–B:84–86. 10.1302/0301-620X.96B11.34305 25381415

[25] Salaffi F , Stancati A , Silvestri CA , Ciapetti A , Grassi W . Minimal clinically important changes in chronic musculoskeletal pain intensity measured on a numerical rating scale. Eur J Pain. 2004;8:283–291. 15207508 10.1016/j.ejpain.2003.09.004

[26] Schindler OS . The controversy of patellar resurfacing in total knee arthroplasty: Ibisne in medio tutissimus? Knee Surg Sports Traumatol Arthrosc. 2012;20:1227–1244. 22484417 10.1007/s00167-012-1985-7 PMC3378836

[27] Schulz KF , Altman DG , Moher D . CONSORT 2010 statement: updated guidelines for reporting parallel group randomised trials. BMJ. 2010;340:c332. 20332509 10.1136/bmj.c332 PMC2844940

[28] Scuderi GR , Bourne RB , Noble PC , Benjamin JB , Lonner JH , Scott WN . The new Knee Society Knee Scoring system. Clin Orthop Relat Res. 2012;470:3–19. 22045067 10.1007/s11999-011-2135-0 PMC3237971

[29] Shekhawat D , Singh A , Banerjee MK , Singh T , Patnaik A . Bioceramic composites for orthopaedic applications: a comprehensive review of mechanical, biological, and microstructural properties. Ceram Int. 2021;47:3013–3030.

[30] Tang XB , Wang J , Dong PL , Zhou R . A meta‐analysis of patellar replacement in total knee arthroplasty for patients with knee osteoarthritis. J Arthroplasty. 2018;33:960–967. 29191443 10.1016/j.arth.2017.10.017

[31] Teel AJ , Esposito JG , Lanting BA , Howard JL , Schemitsch EH . Patellar resurfacing in primary total knee arthroplasty: a meta‐analysis of randomized controlled trials. J Arthroplasty. 2019;34:3124–3132. 31427130 10.1016/j.arth.2019.07.019

[32] Watanabe T , Koga H , Horie M , Katagiri H , Sekiya I , Muneta T . Post‐cam design and contact stress on tibial posts in posterior‐stabilized total knee prostheses: comparison between a rounded and a squared design. J Arthroplasty. 2017;32:3757–3762. 28780225 10.1016/j.arth.2017.07.010

[33] Watanabe T , Koga H , Katagiri H , Otabe K , Nakagawa Y , Muneta T , et al. Coronal and sagittal laxity affects clinical outcomes in posterior‐stabilized total knee arthroplasty: assessment of well‐functioning knees. Knee Surg Sports Traumatol Arthrosc. 2020;28:1400–1409. 30980120 10.1007/s00167-019-05500-8

[34] Watanabe T , Muneta T , Koga H , Horie M , Nakamura T , Otabe K , et al. In‐vivo kinematics of high‐flex posterior‐stabilized total knee prosthesis designed for Asian populations. Int Orthop. 2016;40:2295–2302. 27038027 10.1007/s00264-016-3176-5

[35] Wimmer MA , Pacione C , Yuh C , Chan YM , Kunze J , Laurent MP , et al. Articulation of an alumina‐zirconia composite ceramic against living cartilage ‐ an in vitro wear test. J Mech Behav Biomed Mater. 2020;103:103531. 31756562 10.1016/j.jmbbm.2019.103531

[36] Zheng Y , Li Y , Yuan Z , Geng X , Tian H . Comparison of the accuracy and efficacy of different assistive techniques in primary total knee arthroplasty: a network meta‐analysis. J Exp Orthop. 2024;11:e70098. 39619732 10.1002/jeo2.70098 PMC11604599

[37] Zmistowski BM , Fillingham YA , Salmons HI , Ward DT , Good RP , Lonner JH . Routine patellar resurfacing during total knee arthroplasty is not cost‐effective in patients without patellar arthritis. J Arthroplasty. 2019;34:1963–1968. 31104838 10.1016/j.arth.2019.04.040

